# Inter-surgeon Variability in Cystic Artery Lymph Node Excision during Laparoscopic Cholecystectomy

**DOI:** 10.7759/cureus.2759

**Published:** 2018-06-07

**Authors:** Arkadiusz P Wysocki, Skyle Murphy, Ingrid Baade

**Affiliations:** 1 Department of Surgery, Logan Hospital; 2 Griffith University Medical School, Griffith Health Centre; 3 Queensland Cyber Infrastructure Foundation Ltd, University of Queensland

**Keywords:** laparoscopic cholecystectomy, lymph nodes, safety, operative technique

## Abstract

Introduction

Expert opinion recommends that surgeons perform a laparoscopic cholecystectomy (LC) in a standardized manner by dissecting the hepatobiliary triangle lateral to the cystic artery lymph node (LN) to minimize the rate of a major bile duct injury.

Methods

To determine whether surgeons performed a laparoscopic cholecystectomy in a standardized manner, the study assessed the variability in the frequency of an LN excision. All LCs that were performed at a single hospital were identified from a prospective dataset. The presence of an LN was retrospectively determined from the histology report.

Results

Twenty-seven surgeons were recorded to have performed 2332 laparoscopic cholecystectomies. Out of the total number of patients, 76.8% were female. The median patient age was 42.4 years. About 60.8% of the LCs were elective, while 39.2% of them were acute. Nineteen pathologists reported that in 99% of the specimens – the LN status of 1831 (78.5%) gallbladders was reported and analyzed. Overall, the LN yield per surgeon varied from 0% to 50% (mean 18.7%).

Conclusion

The high inter-surgeon variability in the rate of LN excision during laparoscopic cholecystectomy shows that surgeons dissect the hepatobiliary triangle differently. The LN yield may also represent a surrogate marker of surgical technique (which is easy to measure).

## Introduction

Expert opinion [1] recommends that surgeons routinely dissect the hepatobiliary triangle lateral to the cystic artery lymph node (LN) during laparoscopic cholecystectomy (LC) in order to minimize the rate of a bile duct injury. In this "standardized method" [1] for LC, the closer the surgeon dissects to the gallbladder (and thus away from the bile duct), the less frequently the LN would be excised and, theoretically, the less frequently the bile duct would be injured. By excising the lymph node (whether intentionally or otherwise), some part of the dissection would have been medial to the node and, thus, closer to the bile duct. There is certainly an equipoise regarding this concept because, on the other hand, it is outrightly rejected by some surgeons and has received little attention in the surgical literature.  

Because an LC is typically performed for a benign condition, the excision of the node overlying the cystic artery is incidental i.e., it is not medically necessary. In a recent study, it was found that the LN was excised in 10.4% of 1,332 consecutive LCs by a single surgeon [2]. In that report, the rate of LN excision was independent of most patient factors and the degree of surgical difficulty as assessed using the non-validated Nassar classification [3], suggesting that the rate of LN excision is more a marker of surgical technique rather than being determined by anatomy or pathology.

This retrospective study was designed to evaluate the rate with which the LN was excised by surgeons performing laparoscopic cholecystectomy in a single hospital. The inter-surgeon variability would indicate that surgeons do not dissect the hepatobiliary triangle during laparoscopic cholecystectomy in a standardized manner. If the awareness of the importance of not dissecting medial to this node varies between surgeons, then the rate of LN excision would be expected to differ and would thus represent an easily measurable marker of surgical technique. Due to the rarity of a bile duct injury (BDI), and the relatively small sample size, it is well beyond the scope of this study to determine whether a relationship exists between the rate of LN excision and surgical skill, the safety of dissection, complications, or bile duct injury.

## Materials and methods

All the LCs were identified from the operating room management information system (ORMIS). The surgeons performed the LCs in their usual manner and the technique used could not be influenced by this retrospective study. Pathologists reported the specimens in their usual manner as well and were not routinely instructed to specifically comment on the LN or its presence. The presence of the LN was retrospectively determined by reviewing the original histology report of the resected specimen. The gallbladder specimen was not re-examined by a pathologist for the purposes of this study.

Demographic data were manually extracted from the medical record: the patient's age, sex, and the timing of the surgery (elective vs emergency) were recorded. The operative notes in the ORMIS were of inadequate quality to determine the severity of the Nassar disease and operative difficulty [3]. The operating time was also determined from the ORMIS where theatre nursing staff would routinely record the procedure start and finish times.

LN status was classified as: present (LN mentioned in the macroscopic or microscopic histological description), absent (LN described by the pathologist as “not seen”), or unknown (no mention of LN in pathology report). Known LN status was defined as LN present + LN absent. LN yield was defined as LN present as a percentage of the known LNs. Operations where the LN status was unknown were also reported but were excluded from analysis rather than making the assumption that the unknown LN status was equivalent to LN absent.

Data were stored using Microsoft Excel (Microsoft, Redmond, Washington) and analyzed using R (R Foundation for Statistical Computing, Vienna, Austria). The LN yield was modelled through a logistic regression as a function of three categorical variables (timing of surgery, sex, and the surgeon) and one continuous variable (patient age). The operating time was regarded as being controlled by the surgeon, already represented as a categorical variable in the model. A fast backward elimination of the factors from a logistic regression model was used to identify which variables were important in explaining the LN yield [4]. The procedure was implemented in R with the fastbw function [5]. The fastbw procedure was also used to determine which of the variables, i.e., age, timing of surgery, sex, surgeon and age/surgeon interaction, were necessary to construct a model to adequately explain the LN yield.

## Results

This prospective clinical case series includes all the laparoscopic cholecystectomies performed at Logan Hospital in Queensland, Australia, between January 2011 and August 2016. In Total, 2,332 laparoscopic cholecystectomies were performed by 27 surgeons (surgeons designated as S01 – S27). The cystic artery lymph node was histologically identified in 342 specimens (18.7% of LC with known LN status i.e., 342/1831). The mean number of cholecystectomies per surgeon was 86 (2332/27). Each surgeon’s frequency of LN excisions is presented in Table 1. The rate of LN excision varied from 0% (six surgeons who together performed 59 LCs – 2.5% of all LC) to 50.0% (one surgeon who performed 7 LCs – 0.3% of all LC). The interquartile range for patients’ ages was 29.9 – 54.5 years. Ten percent of the ages recorded  were less than 24.3 years and 10% were greater than 66.8 years.

**Table 1:** Characteristics of each surgeon’s patients Surgeons are listed in order of increasing lymph node yield. LC: Laparoscopic cholecystectomy; LN: Lymph node * Surgeon S03 had 19 missing values for operating time

Surgeon	Number LC	LN status known (%)	LN yield (% of known)	Acute LC (%)	Female (%)	Median operating time (mins)	Median age (years)
S22	3	3 (100.0%)	0 (0.0%)	3 (100.0%)	2 (66.7%)	84	55.5
S11	6	4 (66.7%)	0 (0.0%)	1 (16.7%)	3 (50.0%)	80	34.5
S04	9	4 (44.4%)	0 (0.0%)	5 (55.6%)	7 (77.8%)	67	54.5
S06	11	11 (100.0%)	0 (0.0%)	8 (72.7%)	9 (81.8%)	92	47.4
S15	28	20 (71.4%)	0 (0.0%)	0 (0.0%)	21 (75.0%)	89	49.1
S23	2	0 (0.0%)	0 (N/A)	2 (100.0%)	2 (100.0%)	50	39.5
S08	50	36 (72.0%)	1 (2.8%)	47 (94.0%)	36 (72.0%)	81	44.8
S16	91	78 (85.7%)	7 (9.0%)	28 (30.8%)	69 (75.8%)	60	43.4
S05	11	11 (100.0%)	1 (9.1%)	3 (27.3%)	6 (54.5%)	78	42.9
S19	89	86 (96.6%)	9 (10.5%)	34 (38.2%)	68 (76.4%)	99	41.4
S20	44	42 (95.5%)	5 (11.9%)	36 (81.8%)	36 (81.8%)	87	46.3
S02	38	20 (52.6%)	3 (15.0%)	37 (97.4%)	24 (63.2%)	97	38.9
S10	64	64 (100.0%)	10 (15.6%)	14 (21.9%)	49 (76.6%)	86	45.2
S27	713	510 (71.5%)	80 (15.7%)	166 (23.3%)	569 (79.8%)	71	41.8
S17	93	88 (94.6%)	14 (15.9%)	37 (39.8%)	75 (80.6%)	79	39.9
S14	88	86 (97.7%)	14 (16.3%)	23 (26.1%)	66 (75.0%)	83	43
S07	19	18 (94.7%)	3 (16.7%)	14 (73.7%)	15 (78.9%)	100	44.7
S18	7	5 (71.4%)	1 (20.0%)	3 (42.9%)	3 (42.9%)	73	33
S21	109	60 (55.0%)	13 (21.7%)	61 (56.0%)	80 (73.4%)	83	42.1
S24	178	120 (67.4%)	26 (21.7%)	59 (33.1%)	133 (74.7%)	63	42.6
S13	129	110 (85.3%)	24 (21.8%)	108 (83.7%)	94 (72.9%)	71	40.9
S26	74	41 (55.4%)	10 (24.4%)	70 (94.6%)	55 (74.3%)	88	41.6
S09	16	15 (93.8%)	4 (26.7%)	16 (100.0%)	15 (93.8%)	90	36.7
S03	155	137 (88.4%)	38 (27.7%)	39 (25.2%)	120 (77.4%)	46*	42.9
S12	280	248 (88.6%)	73 (29.4%)	80 (28.6%)	215 (76.8%)	61	43.3
S01	18	12 (66.7%)	5 (41.7%)	18 (100.0%)	13 (72.2%)	86	38.8
S25	7	2 (28.6%)	1 (50.0%)	3 (42.9%)	6 (85.7%)	75	37.3
TOTAL / MEAN	2332	1831 (78.5%)	342 (18.7%)	915 (39.2%)	1791 (76.8%)	73	42.4

**Figure 1:**
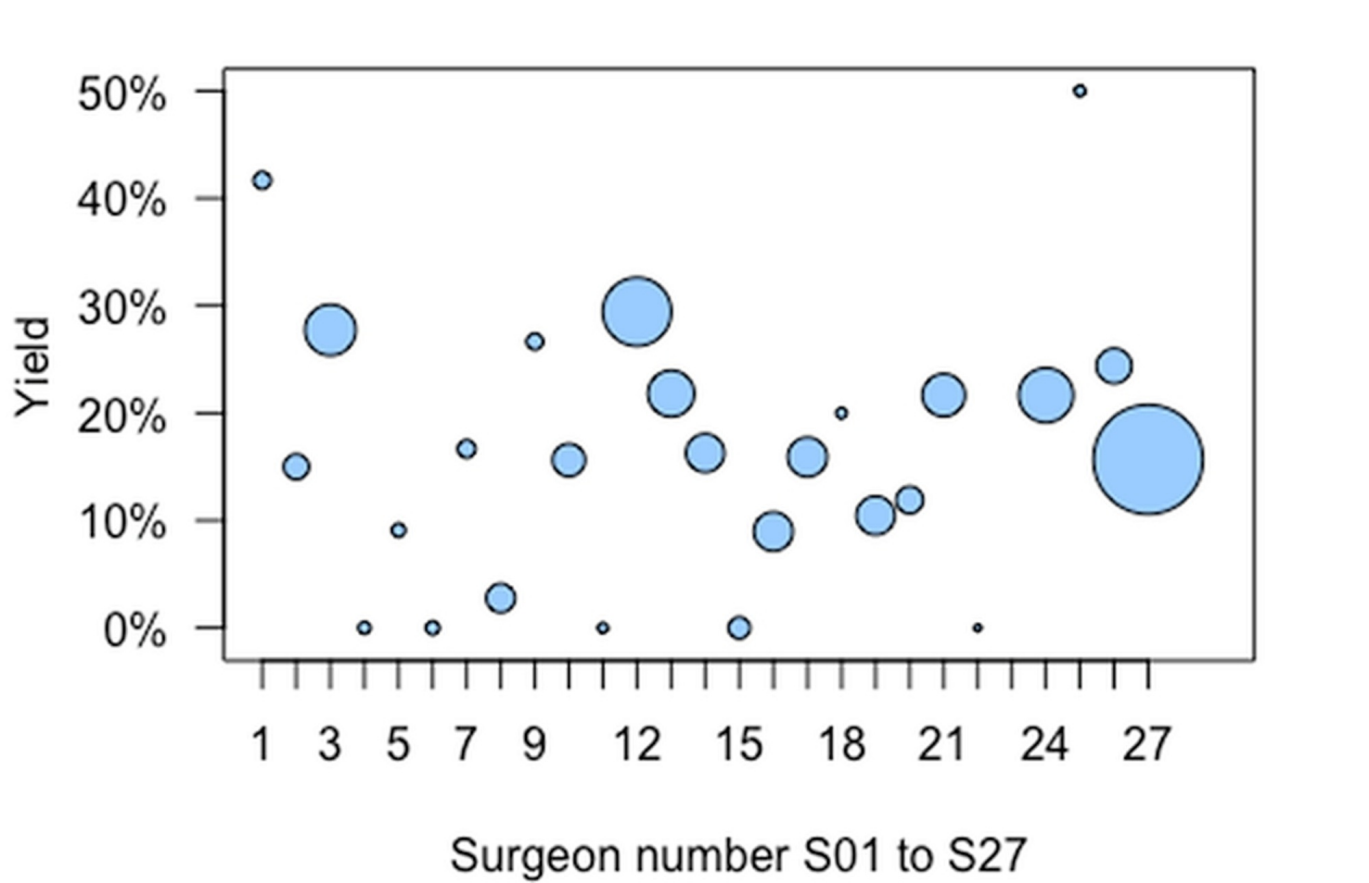
Each surgeon’s (S01 to S27 on horizontal axis) LN yield (vertical axis) Bubble surface area represents the number of laparoscopic cholecystectomies performed e.g. S08 = 50 or bubble S27 (n = 713) is 2.5 times larger than S12 (n = 280). LN: Lymph Node

Several surgeons were excluded from the analysis of LN yield as they only performed a small number of LCs, as indicated by the small bubble areas in Figure 1. Surgeon 23 performed two LC and the LN status was not reported for either. Surgeons 4, 6, 11, 15, and 22 performed 59 LCs in total (2.5% of LCs). But the LN status was known for only 42 of these 59 surgeries, and in each case, the LN was not present (shown as bubbles at 0% yield in Figure 1). A further seven surgeons were excluded from the analysis because they performed a low number of LCs which would make fitting the logistic regression model problematic. These surgeons, 1, 2, 5, 7, 9, 18, and 25 performed 116 LCs (5.0% of total) with an LN yield of 18 from the 83 LCs where the LN was reported (21.7%). One further surgeon, surgeon 8 (50 LCs, 2.1% of all LCs), was excluded because their data was atypical, with a yield of 1 LN present out of 36 reported.

The remaining 13 surgeons performed between 41 and 510 LCs for which the LN status was reported. These surgeons' yields varied from 9.0% (surgeon 16, 7/78) to 29.4% (surgeon 12, 73/248) – a 3.3 fold variation.

**Figure 2:**
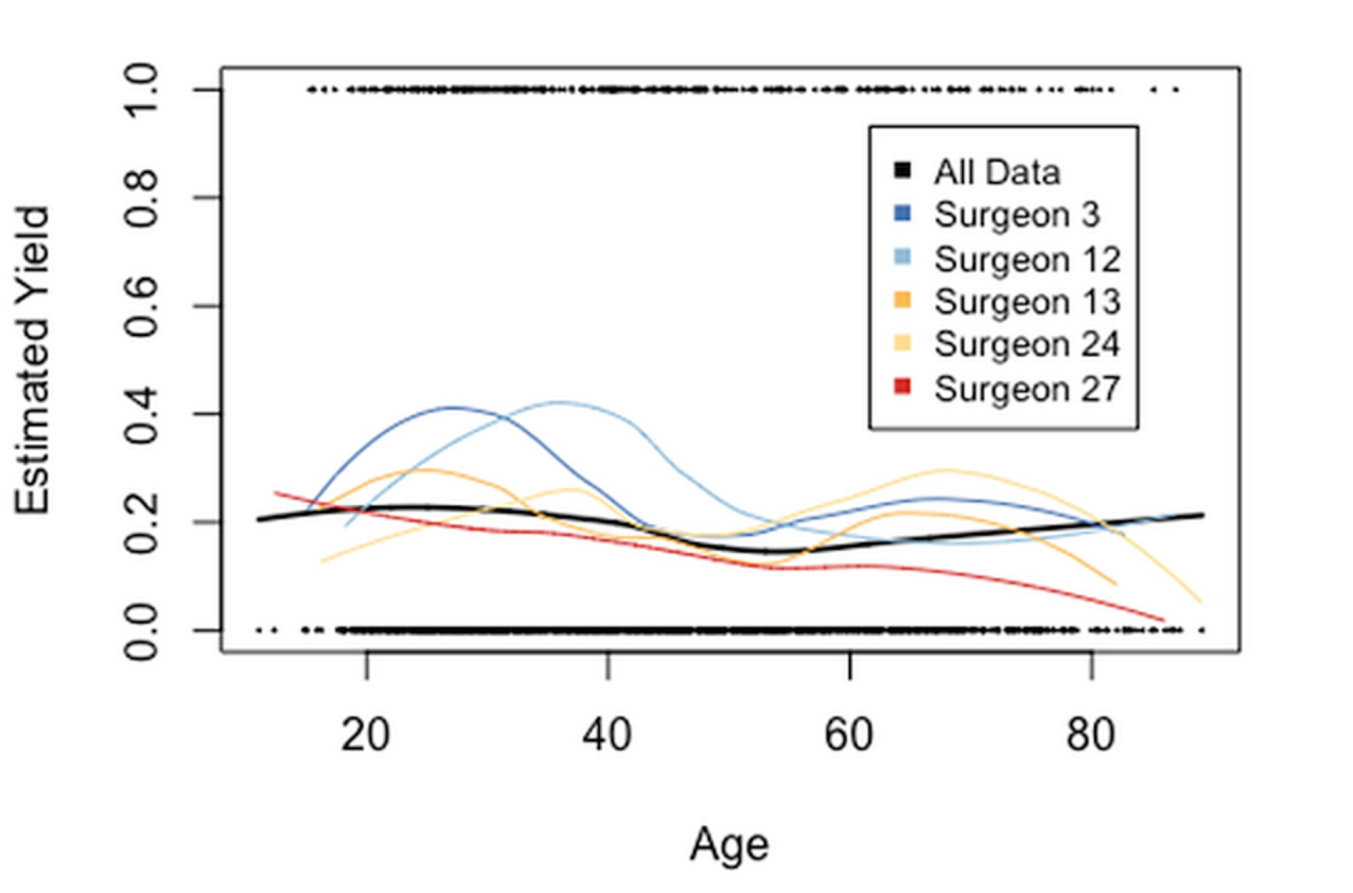
Local regression smooth fitted to LN binary data Cumulative data shown for all surgeons as well as individual data for the five surgeons with the greatest numbers of surgeries. The ages of patients who had LN present are shown across the top of the plot and who had LN absent across the bottom of the plot. Estimates of yield in the tails of the age distribution have high variability. LN: Lymph Node

**Figure 3:**
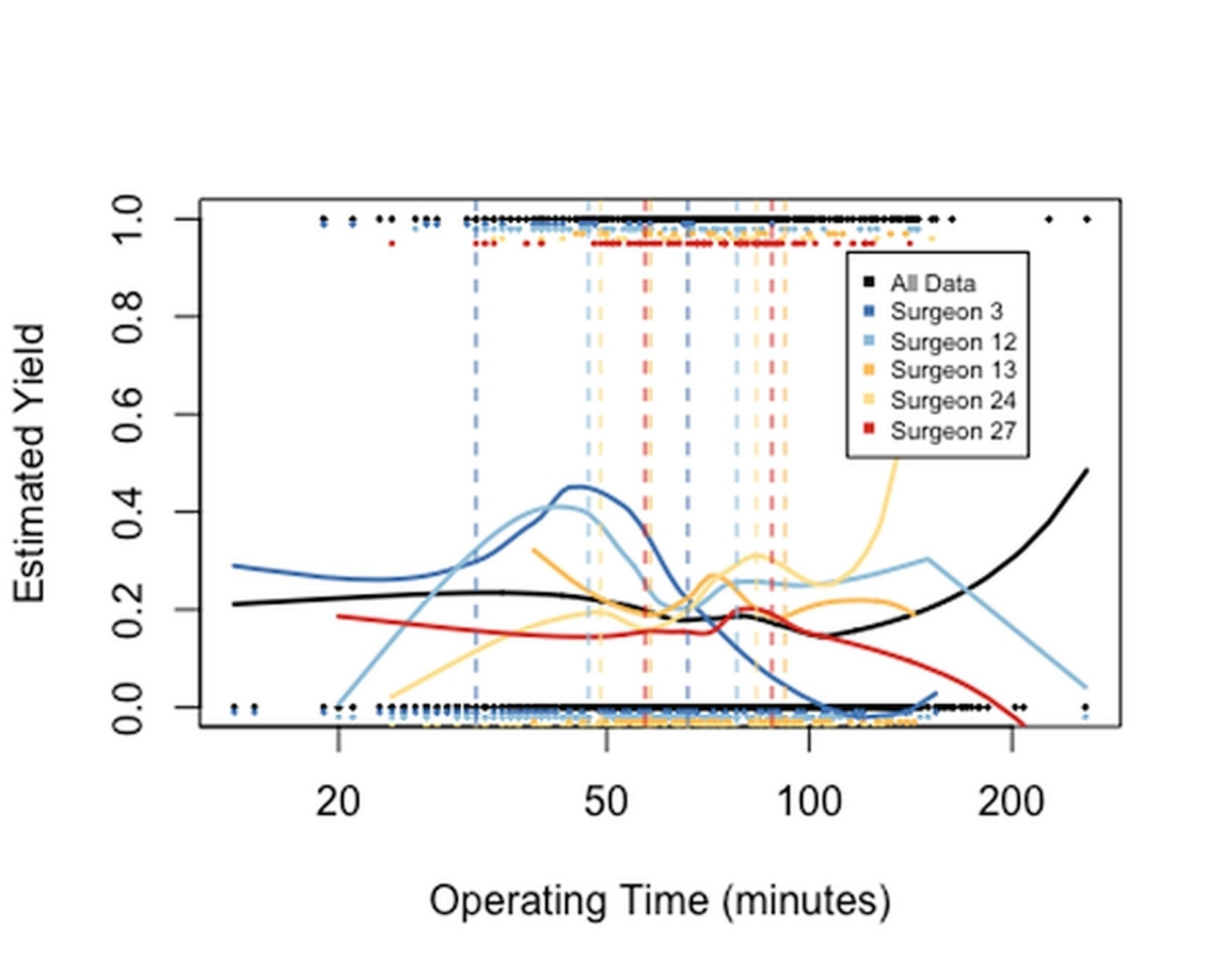
Local regression smooth fitted to LN binary data for all surgeons, and for the five surgeons with the greatest numbers of surgeries The operating time for patients who had LN present are shown across the top of the plot and who had LN absent across the bottom of the plot. Estimates of yield in the tails of the operating time distribution have high variability. LN: Lymph Node

An interaction between the patient's age and the surgeon was also included in the model. The interaction term was suggested by LOcal regrESSion (LOESS) smooths of the age variable for the five surgeons who had the largest numbers of LCs. The LOESS smooths, shown in Figure 2, estimate the LN yield over the range of patient ages by a weighted least squares method. The estimating curves for the surgeons are not simply vertical shifts of identical curves, suggesting that the effect of a patient's age on the LN yield differs between surgeons. Smooths for the timing of surgery and gender are similar to the smooths for all data shown in black in Figure 3. So, the variables timing of surgery and gender are not fitted with an interaction with age. The LOESS smooths estimating the LN yield over each surgeon’s range of operating times is shown in Figure 3, because the wide spread is interesting (the operating time is a surgeon characteristic rather than a patient demographic).

There were significant differences in the LN yield among the surgeons (X2 = 54.7, p < 0.001). In addition, the LN yield varied with patient age, with older patients less likely to have an LN present (X2 = 26.9, p = 0. 0128). There was also a significant interaction between the surgeon and  age in determining the LN yield (X2 = 21.5, p = 0. 0440). The variables surgical timing (acute or elective, X2 = 0.4, p = 0. 5486) and patient sex (X2 = 3.2, p = 0. 0725) were less important in determining the LN yield. The fastbw procedure resulted in a model where the surgeon was the only variable necessary to explain the LN yield in a parsimonious fashion.

**Figure 4:**
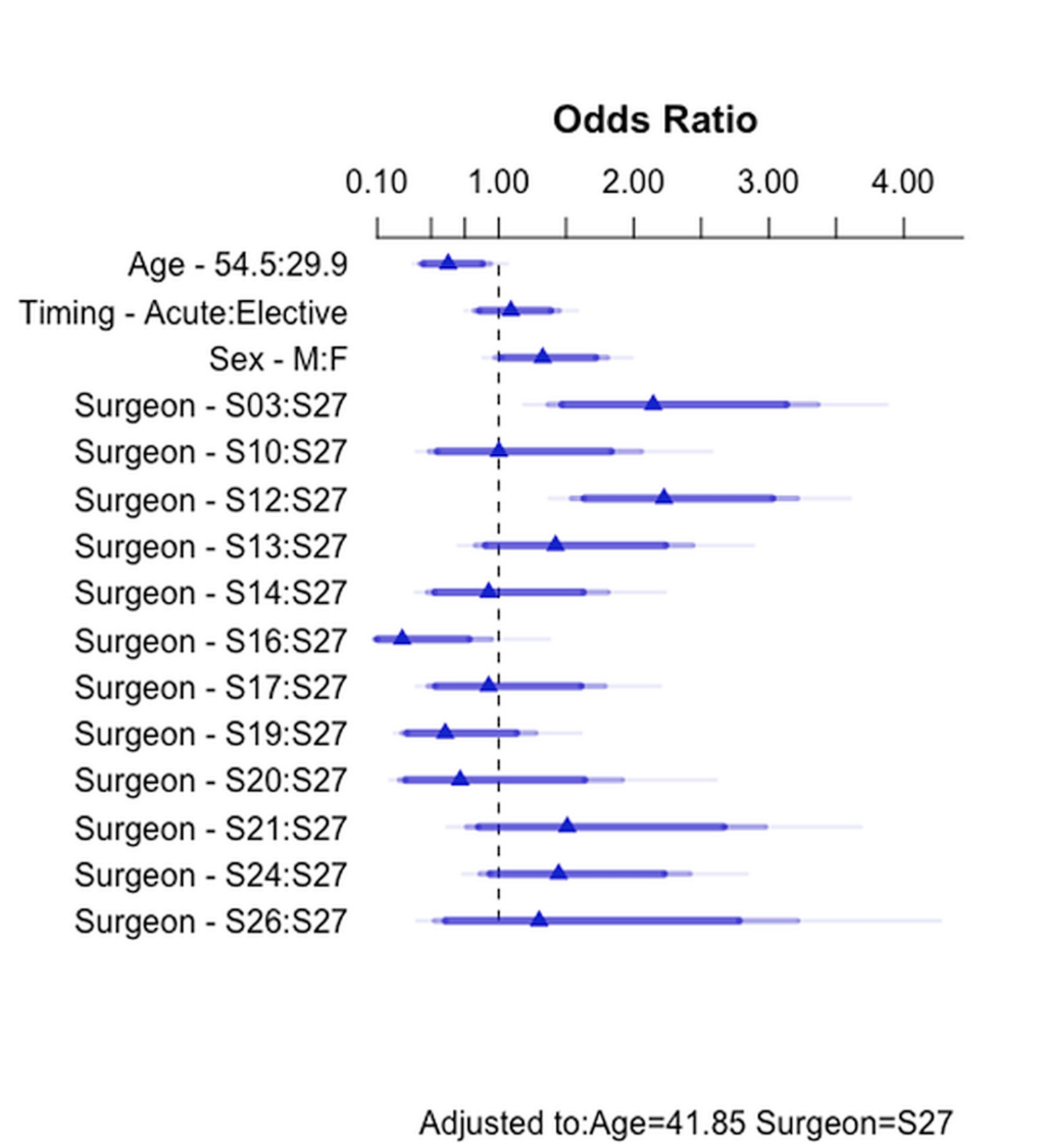
The interquartile range odds ratio for the continuous predictor age compares the 3rd quartile with the 1st quartile of age Simple odds ratios for the categorical predictors timing, sex and surgeon compare each group with the reference group (the largest group). Darkest bars indicate 90% confidence intervals and lighter bars indicate 95% and 99% confidence intervals.

For this model, the odds ratios are shown in Figure 4. The estimates of the surgeons’ LN excision odds ratios varied by a factor of 7.8, from 0.286 for surgeon 16 to 2.225 for surgeon 12. While estimates 0.286 and 2.225 are in comparison to surgeon S27 who performed the greatest number of laparoscopic cholecystectomies, the factor of 7.8 compares the highest rate of lymph node excision (S12) to the lowest rate of LN excision (S16).

## Discussion

The rate of cystic artery lymph node excision during a laparoscopic cholecystectomy varied widely between surgeons, demonstrating a lack of standardization of hepatobiliary triangle dissection. The mean LN yield was 18.7%, but the range was broad (0% to 50%). For the 13 surgeons included in the statistical analysis, the yield varied more than three-fold (from 9% to 29.4%). This project provides no evidence as to why such inter-surgeon variability should exist, but we propose that it may stem from a lack of acceptance or awareness of the importance of the LN from an operative safety perspective. The Royal College of Pathologists of Australia (RCPA) recommends routine documentation of the number, site, and maximal diameter of all lymph nodes only in laparoscopic cholecystectomy specimens where malignancy is present [6]. By excluding all the histology reports where the LN status was not explicitly reported (21.5% of all LC), we have found that the surgeon was the key predictor of LN excision.

**Figure 5:**
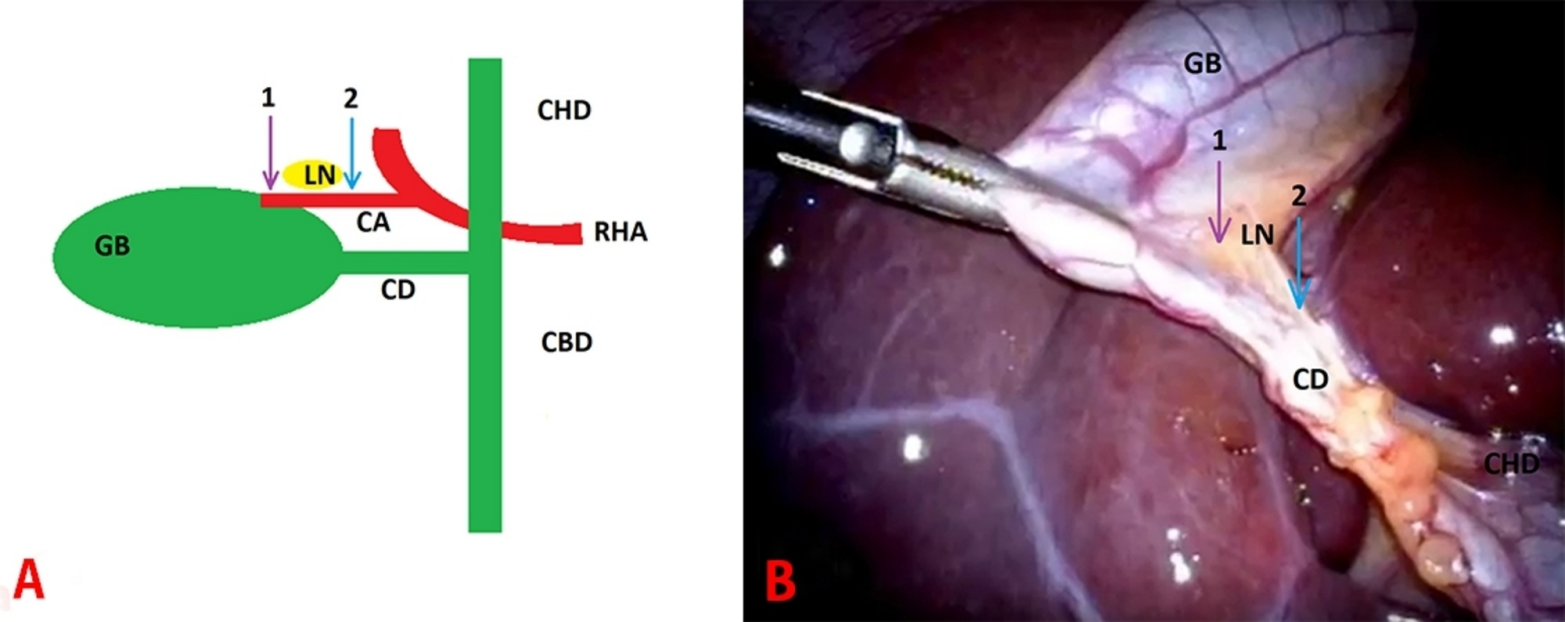
A. diagrammatic representation of gallbladder anatomy. B. Operative image. GB: gallbladder; LN: lymph node; CD: cystic duct; CA: cystic artery; CBD: Common bile duct; CHD: common hepatic duct; RHA: right hepatic artery; arrow 1: dissection lateral to LN; arrow 2: dissection medial to LN. Operative image courtesy of Dr Ehab Akkary

One part of the standardized method for laparoscopic cholecystectomy proposed by Connor et al. calls for dissection “close to the gallbladder and lateral to the cystic node”, in order to reduce the prevalence of major bile duct injury [1]. This concept is illustrated diagrammatically in Figure 5A. The authors were unable to find other literature to support this expert recommendation. The two most recent laparoscopic cholecystectomy guidelines make no mention of the lymph node [7-8], possibly due to lack of recognition, awareness, acceptance or evidence of its role from a safety perspective.

Currently, surgeons have minimized the rate of bile duct injury by thoughtful dissection (key common sense concept termed critical view of safety or CVS [9]) and performing an intraoperative cholangiogram (IOC). There is a lack of level 1 evidence to support both the approaches. A recent review of population-based studies concluded that IOC use is a marker of surgical technique [10], i.e., it is not independent of the surgeon. Somewhat surprisingly, in one in four LCs the surgeon recorded in the operation note that CVS was achieved, this could not be corroborated from the video recording [11]. To differentiate between the role of the IOC from the surgeon performing the cholangiogram (and CVS), an independent and easy way to measure a marker of surgical technique would be useful. A positive aspect of this paper may be to raise surgeons' awareness of another unproven anatomical landmark which may theoretically improve the safety of dissection, such as Rouviere's sulcus or the shape of the quadrate lobe's inferior surface.

The rate at which a surgeon incidentally excises the cystic artery lymph node represents a simple way to measure a marker of their surgical technique. Striving for a low rate of cystic artery lymph node excision may represent a way to improve the standards of clinical quality, i.e., ‘Getting it Right the First Time’ (GIRFT). However, a high LN yield does not necessarily imply that the surgeon is unskilled, because some surgeons may choose to dissect out the node in order to identify the cystic artery beneath it, while others may open the hepatobiliary triangle medial to the LN (and thus excise the LN) as this allows access to the cystic artery prior to its bifurcation, allowing for a quicker dissection. Under other circumstances, e.g., acute cholecystitis, the surgeon may choose to commence the dissection high on the gallbladder (i.e., lateral), thus dividing multiple cystic artery branches and not excising the lymph node. It is well beyond the scope of this study to determine whether a relationship exists between the rate of LN excision and surgical skill, the safety of dissection, complications, or bile duct injury. A relationship between a surgeon's LN yield and a major BDI rate could be determined with a population-based dataset such as the Swedish national quality registry called GallRiks.

Sometimes, the lymph node is visible as a bulge through the peritoneum before any dissection has taken place. It typically becomes visible after opening the peritoneum at the gallbladder–hepatobiliary triangle junction as shown in Figure 5B above. The standardized method then recommends that the surgeon continues the hepatobiliary triangle dissection lateral to the lymph node. There is no requirement to search for or dissect out the cystic artery node – in fact, doing so may result in the dissection being too medial. In a recent study, the LN was identified by pathologists in 10.4% of 1,332 consecutive laparoscopic cholecystectomies by a single surgeon [2]. On multivariable analysis, only three out of the 19 variables studied were associated with a lower rate of LN excision (presence of a senior surgical trainee, American Society of Anesthesiologists class 3, and spillage of gallstones). Otherwise, the rate of LN excision was independent of disease severity and surgical difficulty as assessed by the Nassar disease severity and operative difficulty scales [3]. An LN excision rate of 0% is probably not achievable for anatomical (e.g., small not visible node, short cystic duct or artery or multiple lymph nodes) or pathological (e.g., bulky abnormal appearing node) reasons.

Typically, the cystic artery travels through the hepatobiliary triangle (82% in a recent literature review [12]). The LN is not always visible as it is within the fat, deep in the peritoneum, and varies in size. According to Skandalakis, “the lymph node of Calot (sic) usually lies just superficial to the position of the cystic artery in the cystic triangle (sic) and can be a good guide to finding and ligating it” [13]. The anatomists go on to state that “cystic node… lies in the angle formed by the cystic and common hepatic ducts” [13].

In Qamar et al., the cystic artery lymph node was found to be always on top of the cystic artery (i.e., superficial) – in 59% it was anterior and in 41% posterior to the artery [14]. The node may not be solitary and uniformly present first reported some 50 years ago by Williams and Whittaker in 80 postmortem examinations [15]. A triple cystic artery lymph node has been reported [16]. Similar to our cystic artery LN excision rate of 18.7%, Katri et al. intraoperatively identified the LN in 85.7% of LCs (126/147) and excided the LN in 20.6% (26/126) [17]. However, Qamar et al. intraoperatively identified the LN in only 53% of LC (160/300) [14]. Channa et al. intraoperatively identified the LN in 78.3% of LCs (170/217) and the surgeon excised the LN in 54.7% [18]. Such variability of LN excision shows differences in surgical technique and an awareness of the lymph node's importance from a safety perspective. Excision of the LN is an emerging marker of surgical technique [2].

The strength of the study is its uniqueness and the large number of patients having surgery within a short period of time. Comparing the rate of LN excision amongst 27 surgeons operating at a single hospital means that the results are generalizable. By excluding the histology reports where the LN status was unknown (only 21.5% of LC), the study made no assumptions as to the presence or absence of the LN. Being retrospective is typically a research limitation; however, in this instance it is a major strength as surgeons could not vary their technique i.e., avoiding the observer effect (Hawthorne effect) [19] and artificially avoid excising the LN. A prospective study to assess LN excision will be difficult to interpret because once the surgeon is made aware that LN excision is under study, the surgeon may vary their technique by actively looking for the LN, and upon finding it, changing the dissection trajectory to leave the LN behind.

This study has a number of limitations: disease severity and difficulty of surgery (e.g., as classified by Nassar) could not be determined due to the quality of the operation report. However, intra-surgeon variability has been shown to be low - across a range of pathologies, levels of difficulty, and surgical approaches, the rate of LN excision by a single surgeon was remarkably constant at 10% [2]. Regardless of whether it was excised or not, it is not known whether the LN was seen by the surgeon; however, between 53% [14] and 86% [17] of lymph nodes could be seen by surgeons in other studies. Some surgeons may dissect out the node from its lateral aspect (leaving it attached medially) in order to visualize the underlying cystic artery without excising the LN. The number of cholecystectomies per surgeon may not be reflective of that surgeon’s volume of work or experience, as some surgeons would have commenced their practice at the hospital towards the end of their study period while others may operate at other hospitals. Controlling for surgeon case-mix, differences may be incomplete given the limited literature as to which factors influence LN excision. The frequency of multiple cystic artery lymph nodes is likely to be very low but is unknown [16], meaning that a small proportion of the patients may have had multiple nodes where one was left behind while the other excised and thus been classified as LN present. Some lymph nodes may have been purposefully excised, e.g., the surgeon was suspicious of the gallbladder or LN appearance – this number is likely very low.

There are several further research opportunities. The Nassar disease severity and surgical difficulty scale needs to be validated/modified as some surgical aspects are not rated (e.g., stiff fatty liver, liver high above costal margin, bulky omentum, abnormal number or position of the gallbladder). The generalizability of these findings should be established. Ultimately, in order to substantiate expert opinion [1] that the hepatobiliary triangle be routinely dissected lateral to the cystic artery lymph node in order to minimize the rate of major bile duct injury, a similar study on a larger population scale is required.

Just as there is an inadvertent rate of parathyroidectomy during thyroidectomy for anatomical reasons [20], there may be an unavoidable rate of cystic artery LN excision during laparoscopic cholecystectomy. The ideal rate remains to be established and may be lower or greater than the one in five observed in this study. A much larger dataset is required to assess the relationship between the rate of lymph node excision and major bile duct injury.

## Conclusions

Hepatobiliary triangle dissection during laparoscopic cholecystectomy is not performed in a standardized manner as evidenced by a wide range in the frequency with which surgeons excise the lymph node. Irrespective of any relationship between lymph node excision and major bile duct injury, each surgeon’s lymph node excision ratio represents a marker of their surgical technique. The ideal cystic artery lymph node excision ratio remains to be established.
